# A Case of Lung Cavity Incidentally Discovered Following Evaluation for Pulmonary Embolism

**DOI:** 10.7759/cureus.58125

**Published:** 2024-04-12

**Authors:** Christopher T Gabbert, Fariha Bhuiyan, James F Witko

**Affiliations:** 1 Medical School, Sentara Halifax Regional Hospital Clinical Site, Edward Via College of Osteopathic Medicine, Blacksburg, USA; 2 Internal Medicine, Sentara Halifax Regional Hospital, Boston, USA; 3 Pulmonary and Critical Care Medicine, Pulmonology Associates of Southside Virginia, Boston, USA

**Keywords:** pneumonia, lung malignancy, deep vein thrombosis, pulmonary embolism, pulmonary cavitation

## Abstract

Cavitary lung lesions manifest following a wide variety of pathological processes, which are typically delineated as infectious and non-infectious. With respect to noninfectious causes, malignancies are among the most worrisome, while autoimmune and embolic processes are less frequent and less severe in prognosis. While it is important to differentiate between these etiologies, treatment may resort to surgical procedures for both diagnostic and curative intent. This case involves the incidental finding of a cavitary lung lesion following pulmonary embolism evaluation. Following confirmation of deep venous thrombosis and pulmonary embolism, the patient was admitted to the hospital, administered anticoagulants, and monitored for changes in respiratory status. Outpatient follow-up showed vast improvement in the cavity without antibiotic/chemotherapeutic treatment. Embolic events were attributed to Factor V Leiden diagnosis. This manuscript aims to discuss etiologies of lung cavities and how treatment strategies may differ depending on pathological processes and concomitant patient comorbidities. Special attention will be paid to pulmonary cavity evaluation in the acute hospital setting.

## Introduction

Cavitary lung lesions are described radiographically as lucent spaces that can have variable surrounding wall thicknesses and contents depending on etiology [1,2]. When considering wall thickness, this feature alone can give some insight into the cause, as thicker-walled cavities are associated with malignancy and rarely with chronic mycobacterial infections [3]. The fluid content is typically pustular from an infectious source, although cavities have been noted to include excessive bronchial secretions, blood, and liquefied necrosis from other etiologies as well. [3]. Regarding the size and number of cavities seen on imaging, these features alone are not diagnostic and thus require additional evaluation. [1-3].

The modalities typically used to investigate cavitary lung lesions include X-ray and computed tomography (CT), with CT giving more detail [1]. Surgical practices are utilized for the purpose of cytological analysis, biopsy, and curative intent [3].

In order to choose the most appropriate treatment, it is necessary to consider the lesion within the proper clinical and historical context. One approach involves differentiating between infectious and noninfectious causes, with an emphasis on ruling out cancer in the latter category [1]. With regard to infectious etiologies, *Mycobacterium tuberculosis* (TB) is one of the more common causes of cavitary lung lesions, with upper lobe lesions typically presenting in immunocompetent patients [1,2]. When considering pulmonary abscesses, cavities are sometimes, but rarely, seen after community-acquired pneumonia. Necrotizing pneumonias are typically caused by *Klebsiella pneumoniae* and *Staphylococcus aureus*, which are more associated with aspiration and post-viral states, respectively [1,2]. Fungal and parasitic infections may also lead to cavitation and should be evaluated within the context of geographic location and immune status. Furthermore, consideration of systemic infections is warranted, given how septic emboli can also result in cavitary lesions within the lung parenchyma [1,2].

For noninfectious sources, malignancy can lead to cavitation, as tumor growth obstructs airways and prevents adequate gas exchange [4]. The incidence is unclear, although previous studies have suggested that cavities are seen in approximately 11% of lung cancers and are usually associated with primary lesions [5]. When considering metastatic causes, one would expect to find multiple lesions rather than a single, solitary cavity [4]. For other noninfectious causes, cavitary lung lesions are seen in several autoimmune diseases, such as sarcoidosis, and are rarely seen after pulmonary infarcts secondary to pulmonary embolism (PE) [1,6]. Infarctions following PE are estimated to occur approximately 30% of the time, with cavitation occurring in an estimated 7% of infarctions [7,8]. This low incidence is thought to be due to dual blood supply to the lungs, which can allow for better tissue recovery [9]. However, these numbers represent aseptic infarcts. Cavitation can also occur following infection of infarcted tissue [6].

This case involves a patient who experienced a subclinical cavitary lung lesion, which was brought to medical attention following a PE incident. Additionally, this case was complicated by dilated cardiomyopathy. Potential etiologies are addressed within this report, along with medical management adjustments that account for comorbidities. Specific focus will be placed on acute hospital work-up for this patient.

## Case presentation

A 55-year-old female was admitted to the hospital from the pulmonary clinic for further workup of dyspnea and possible PE. She had initially presented two weeks prior to an outpatient family physician with complaints of a several-week history of fevers, chills, appetite changes, cough, dyspnea on exertion, chest pain reproducible with deep inspiration, and mid-back pain. She was treated with a 10-day course of azithromycin for suspected right lower lobar pneumonia. At the time of pulmonary clinic evaluation, the patient reported continued symptoms of cough, dyspnea, and loss of appetite, although she disclosed that her pleuritic-like chest pain, mid-back pain, and fevers had resolved while on antibiotics. The patient additionally endorsed a several-week history of intermittent left calf pain.

The patient’s past medical history was significant for obstructive sleep apnea (OSA), obesity, and new-onset hypertension; the patient had no active medications and had never used a continuous positive airway pressure (CPAP) device. She was a non-smoker, although she experienced secondhand smoke in childhood. The patient described being more sedentary within the past six months following the loss of her father to pancreatic cancer. She had no other significant cancer history or hypercoagulable conditions, personally or within her family.

**Figure 1:**
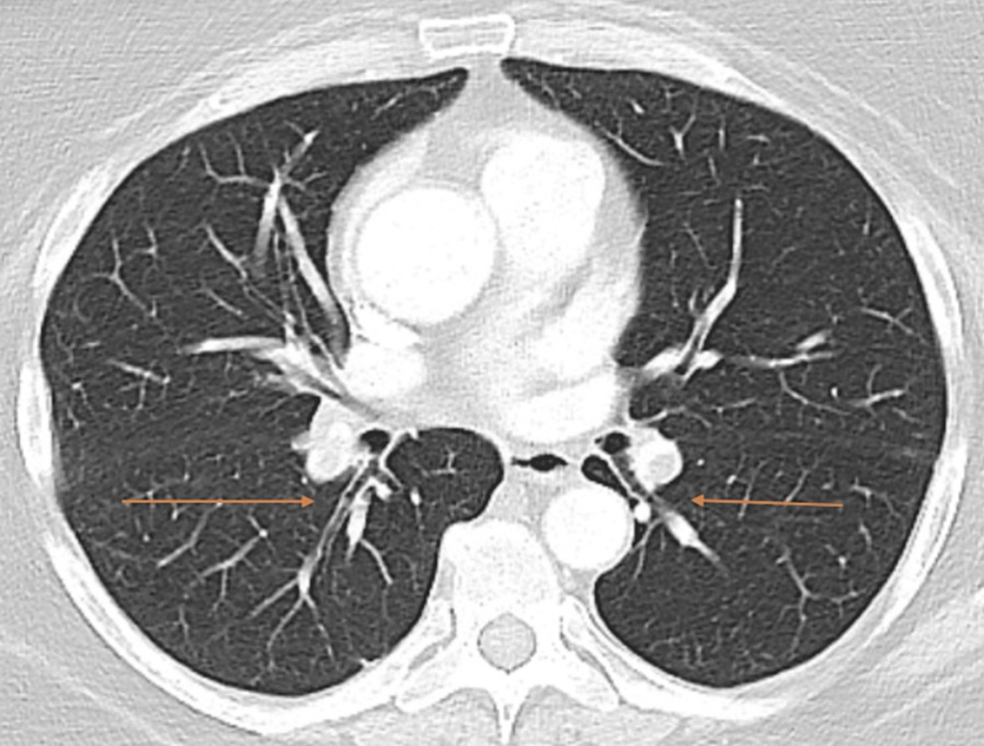
CT angiogram String-like filling defects noted in bilateral posterior pulmonary artery segmental branches.

**Figure 2:**
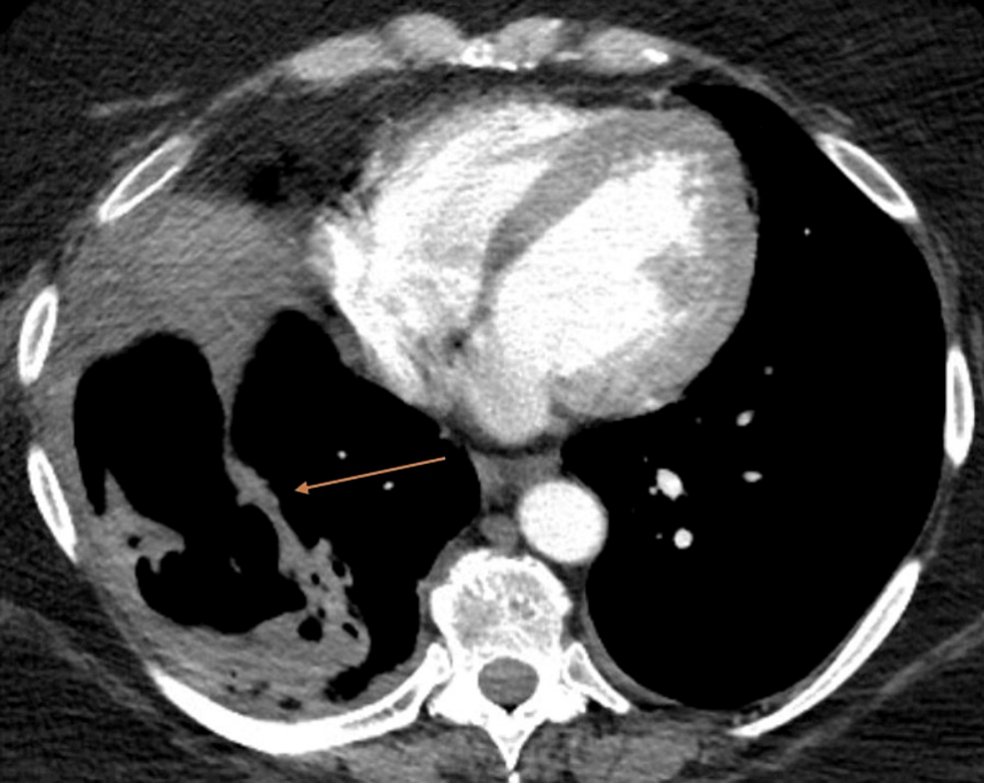
CT angiogram with a cavity in view Right lower lobe cavity seen. Fluid noted posteriorly. Anterior attenuation identified as diaphragm/superior portion of liver.

Upon admission to the hospital, the patient was found to be mildly tachycardic with a heart rate of 104 beats per minute, blood pressure of 146/83 mmHg, respiratory rate of 19 breaths per minute, and pulse oximetry of 94% on room air with a temperature of 97.7℉. She was also found to have a swollen and nontender left calf. D-dimer was elevated at 3.98 mg/L (normal is less than 0.5 mg/L), with ultrasound confirming left lower extremity deep vein thrombosis (DVT). CT angiogram (CTA) of the chest revealed bilateral, segmental pulmonary emboli in the posterior lung bases (Figure 1), which were thought to be subacute, coinciding with her previous history of mid-back pain. No evidence of right heart strain was noted. Additionally, CTA showed a large, thick-walled cavity in the right lung base (Figure 2), along with two nodules found in the left lower lobe, measuring 9 and 12 mm, respectively.

Further lab work revealed a normal white blood cell (WBC) count of approximately 10,000 and a lactic acid value of 1.7 mmol/L. Complete blood count and comprehensive metabolic panel were otherwise unremarkable. The only remaining pertinent lab finding included an elevated N-terminal prohormone of brain natriuretic peptide (NT-proBNP) of 512 pg/mL. Electrocardiography revealed sinus tachycardia.

Following imaging, the patient was initiated on enoxaparin (1 mg/kg every 12 hours) for anticoagulation. An echocardiogram revealed severe left ventricular wall hypokinesis with an ejection fraction of 37%. Pulmonary arterial pressure was indeterminate at the time. The leading diagnosis was Takotsubo cardiomyopathy, which aligned with her reported history of recent emotional trauma following the loss of her father. She was initiated on sacubitril/valsartan (24/26 mg twice daily).

On day 2 of admission, the patient reported feeling less short of breath with minimal changes in cough. Cardiothoracic surgery is recommended after a six-week follow-up chest CT and deferment of potential video-assisted thoracoscopic surgery (VATS). Cardiology recommended outpatient nuclear stress test.

The remainder of her stay was uneventful. She remained hemodynamically stable and described no further symptoms. She was discharged on day three of her hospital stay, with plans for follow-up chest CT, positron emission tomography (PET)/CT to evaluate for malignancy, and cardiac nuclear stress test. She was instructed to continue anticoagulation with rivaroxaban at home, along with metoprolol and sacubitril/valsartan.

**Figure 3:**
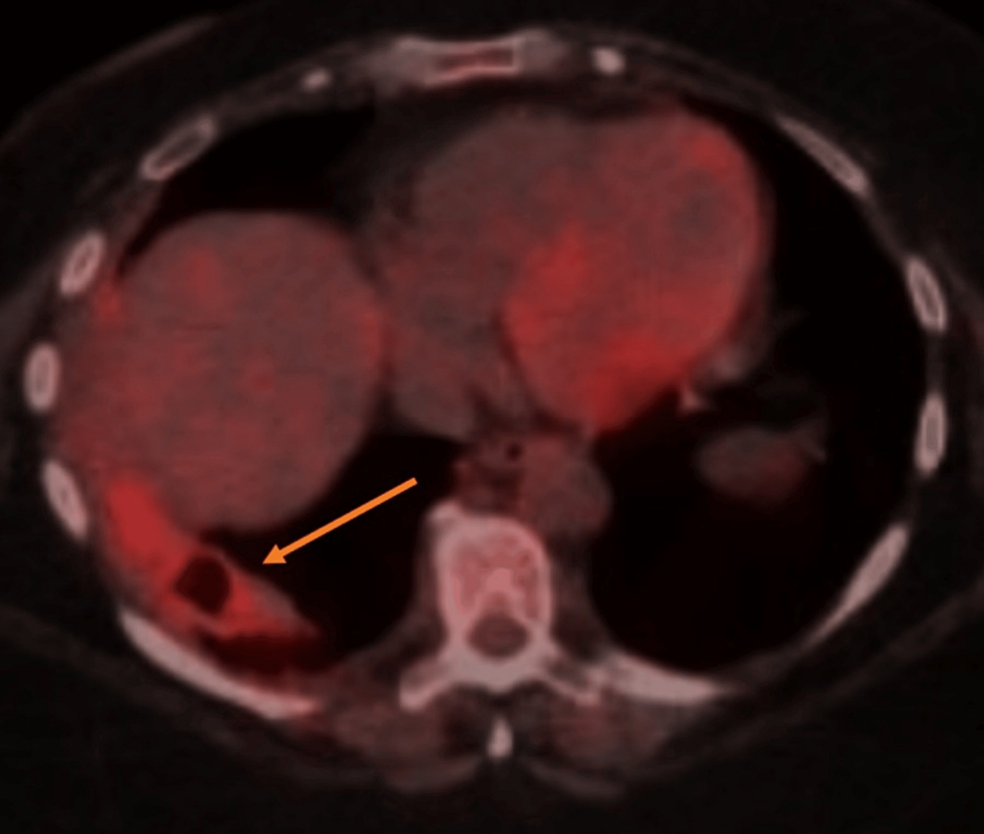
Follow-up PET/CT Cavity decreased in size; radiotracer uptake was noted. Cross-section includes liver. PET, positron emission tomography

**Figure 4:**
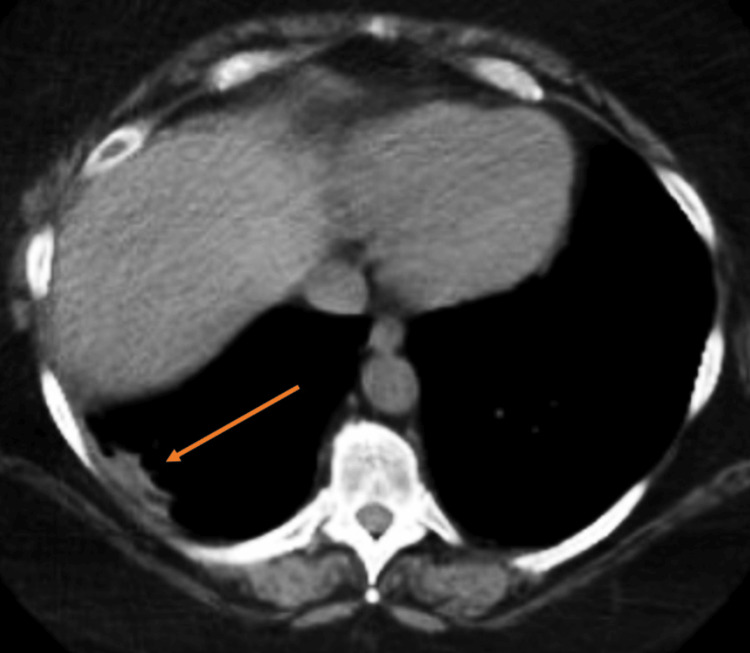
Follow-up CT Marked improvement from previously noted thick-walled cavity.

The patient received a PET/CT study one month after admission, which revealed the cavity to be decreased in size and hypermetabolic, suggestive of an infectious, inflammatory, or malignant process (Figure 3). Both nodules had uptake as well, which was concerning for malignancy. The patient was sent to cardiothoracic surgery, where it was decided that close follow-up was more appropriate than surgical intervention. The most recent imaging was five months after initial admission, which showed a small residual cavity with minimal pleural thickening (Figure 4), along with only one nodule in the left lower lobe measuring 5 mm. The patient continued to do well with outpatient evaluations and reported minimal fatigue. Regular pulmonology follow-up was scheduled until the full resolution of the cavity was achieved.

Regarding her cardiomyopathy, a one-month follow-up nuclear stress test showed reversible inferolateral ischemia with an overall ejection fraction of up to 57%. No medication adjustments were recommended at the time. Furthermore, she had seen hematology/oncology regarding possible hypercoagulable conditions. She was found to be heterozygous for Factor V Leiden. Follow-up with hematology/oncology was pending.

## Discussion

Given the size of the cavity, fluid presence, and the patient’s reported history, an infectious process was initially considered for the cavitary lung lesion. More specifically, the lesion radiographically resembled a bronchopleural fistula with empyema, as fluid was also noted in the pleural cavity. Bronchopleural fistulas most often arise as a result of post-surgical stump failure and the use of positive pressure ventilation [10]. However, pre-existing infections have been noted to form fistulas, including TB and other necrotizing pneumonias [11]. With this patient, TB seemed unlikely given her immunocompetent status, location of cavity, and lack of hilar lymphadenopathy.

Within the general context of lung abscesses, the right lower lobe is a common dwelling place for aspiration pneumonias. The patient’s alcohol history was unknown, and she did not describe any emesis episodes in the weeks leading up to hospital admission; however, her history of sleep apnea could increase her risk of aspiration [12]. It is believed that aspiration can occur as a person attempts to inspire against a closed airway, creating a further negative pressure system, which can draw in pharyngeal contents [12]. Given her lack of CPAP use, this etiology was plausible; however, the definitive diagnosis would have required cytological analysis [1,2].

While hospitalized, the patient was afebrile and showed no obvious signs of active infection, with an unremarkable WBC count and lactic acid. Thus, had the source been truly infectious, the onset would have been insidious, with minimal systemic inflammatory reaction occurring while the cavity enclosed the source. Therefore, the patient was not discharged with antibiotics.

Noninfectious causes were also considered, specifically malignancy. Despite her nonsmoker status, this diagnosis was supported by her secondhand smoke exposure, positive family history of cancer, and the radiographic findings of the cavity wall thickness and lung nodules [1]. Nevertheless, the marked reduction in cavity size, along with the disappearance and reduction of nodules, did not support a malignant cause.

When taking the PEs into account, it seems that the cavitary lung lesion and the embolisms were disjointed. Given the rarity of cavity conversion from a post-PE infarct, it is unlikely that the patient had any previous PE events leading to cavitation [7,8]. However, both infection and malignancy can lead to hypercoagulable states and thus may be common etiologies for both the cavity and PE events [13,14]. This should be considered in subsequent instances, along with utilizing coagulopathy and vessel disease testing to rule out other causes of thrombosis.

Stasis is another plausible cause of this patient’s left calf DVT, given her reported sedentary lifestyle. However, because her activity level cannot be well-objectified, it may have been more fitting to first rule out hypercoagulation as the main cause of thrombus formation.

Regarding the patient’s post-hospital evaluation, it is worth noting that anticoagulation is a relative contraindication for thoracoscopy, the preferred surgical intervention for this patient [15]. This is important, as her recent PE events precluded her from the potential swift evaluation (biopsy) and treatment (drainage) of her lung cavity. In this case, a conservative approach was taken, with subsequent imaging supporting improvement without the need for intervention. Accordingly, deferment of VATS was not detrimental to the patient. Nevertheless, if surgery were indicated, pausing her anticoagulative therapy may have been necessary to minimize the risk of complications [16].

Lastly, the patient was found to have dilated cardiomyopathy on an echocardiogram. While the patient only required medical management and outpatient follow-up, having a low ejection fraction predisposed the patient to worse outcomes if she were to have developed pulmonary venous congestion. Therefore, special attention should be paid to cardiac status in similarly presenting patients, especially within the context of surgical suitability.

## Conclusions

Overall, this case was complicated by many superimposed pathologies. The lung cavity was thought to be most likely secondary to a nonmalignant cause, particularly infection, given how well the cavity and patient symptoms had resolved. Furthermore, the PEs were most likely a result of the patient’s Factor V Leiden diagnosis. Despite this, it remained beneficial to initially consider common etiologies, such as infection and malignancy, that could lead to these temporally distinct events. With regard to the patient’s outpatient evaluation, her anticoagulation use and her cardiomyopathy were both factors that required further attention in the context of pulmonary surgical intervention. Given similar circumstances, patients with multiple cardiopulmonary pathologies requiring distinct regimens must be treated uniquely. We hope that this case can inform future management of patients with superimposed lung pathologies that warrant individualized treatment plans.
